# Changes in Immune Cell Types with Age in Breast are Consistent with a Decline in Immune Surveillance and Increased Immunosuppression

**DOI:** 10.1007/s10911-021-09495-2

**Published:** 2021-08-02

**Authors:** Arrianna Zirbes, Jesuchristopher Joseph, Jennifer C. Lopez, Rosalyn W. Sayaman, Mudaser Basam, Victoria L. Seewaldt, Mark A. LaBarge

**Affiliations:** 1 Department of Population Sciences, Beckman Research Institute, City of Hope National Medical Center, 1500 E Duarte Road, Duarte, CA 91010 USA; 2 Irell and Manella Graduate School of Biological Sciences, City of Hope, Duarte, CA USA; 3 Center for Cancer and Aging, Beckman Research Institute, City of Hope, Duarte, CA USA; 4 Cancer Metabolism Training Program, Beckman Research Institute, City of Hope, Duarte, CA USA; 5 Department of Laboratory Medicine, Helen Diller Family Comprehensive Cancer Center, University of California, San Francisco, San Francisco, CA 94143 USA; 6 Centre for Cancer Biomarkers CCBIO, University of Bergen, Bergen, Norway

**Keywords:** Breast cancer, Prevention, Aging, Mammary gland, Immune milieu, Immunosuppression, Macrophage polarization, Image analysis, Deep learning, Machine learning

## Abstract

A majority of breast cancers (BC) are age-related and we seek to determine what cellular and molecular changes occur in breast tissue with age that make women more susceptible to cancer initiation. Immune-epithelial cell interactions are important during mammary gland development and the immune system plays an important role in BC progression. The composition of human immune cell populations is known to change in peripheral blood with age and in breast tissue during BC progression. Less is known about changes in immune populations in normal breast tissue and how their interactions with mammary epithelia change with age. We quantified densities of T cells, B cells, and macrophage subsets in pathologically normal breast tissue from 122 different women who ranged in age from 24 to 74 years old. Donor-matched peripheral blood from a subset of 20 donors was analyzed by flow cytometry. Tissue immune cell densities and localizations relative to the epithelium were quantified in situ with machine learning-based image analyses of multiplex immunohistochemistry-stained tissue sections. In situ results were corroborated with flow cytometry analyses of peri-epithelial immune cells from primary breast tissue preparations and transcriptome analyses of public data from bulk tissue reduction mammoplasties. Proportions of immune cell subsets in breast tissue and donor-matched peripheral blood were not correlated. Density (cells/mm^2^) of T and B lymphocytes in situ decreased with age. T cells and macrophages preferentially localized near or within epithelial bilayers, rather than the intralobular stroma. M2 macrophage density was higher than M1 macrophage density and this difference was due to higher density of M2 in the intralobular stroma. Transcriptional signature analyses suggested age-dependent decline in adaptive immune cell populations and functions and increased innate immune cell activity. T cells and macrophages are so intimately associated with the epithelia that they are embedded within the bilayer, suggesting an important role for immune-epithelial cell interactions. Age-associated decreased T cell density in peri-epithelial regions, and increased M2 macrophage density in intralobular stroma suggests the emergence of a tissue microenvironment that is simultaneously immune-senescent and immunosuppressive with age.

**Supplementary Information:**

The online version contains supplementary material available at 10.1007/s10911-021-09495-2.

## Background

More than 75% of breast cancers (BC) are diagnosed in women after age 50y [1], however, the mechanisms underlying age-related BC susceptibility are not well understood. The increased incidence is thought to be driven by a combination of accumulated somatic mutations, and changes in the breast tissue microenvironment that unleash malignant cells and increase susceptibility of the tissue to cancer initiation [2]. Age-dependent tissue composition changes in breast include decreased connective tissue, increased adipose cell proportions [3, 4], reduced proportions of myoepithelial cells, accumulation of luminal epithelial cells and dysfunctional progenitor cells with a basal differentiation bias [5–7]. Age-related chronic sterile inflammation in peripheral blood and solid tissues is thought to promote, if not cause, many diseases of aging [8, 9]. However, there is limited understanding of how the immune milieu changes in breast with age.

Immune cells can impose potent phenotypic and compositional changes during mammary gland development [10–13]. Mouse models suggest that the innate and adaptive immune cell types exert opposing effects during mammary gland development. Innate immune cells promote mammary gland branching and proliferation of mammary epithelial cells; e.g., TNF-α secreted by macrophages stimulates proliferation of rat mammary epithelial cells [11]. Conversely, adaptive immune cell types are involved in negative regulation of the mammary gland; e.g., interferon gamma (IFN-ɣ) produced by CD4^+^ T helper 1 (Th1) cells inhibits luminal differentiation and thereby inhibits mammary gland organogenesis [13]. In addition to sculpting developmental processes, the immune system is infamous for its role in tumor progression [14], for example tumor-associated macrophages (TAMs) are polarized to an M2-like phenotype, giving them immunosuppressive functions and further facilitating tumor progression and cancer cell proliferation [15].

Whereas aging adversely impacts all people, women with germline mutations in *BRCA1* or *BRCA2* genes are considered specifically high risk for BC, having an average cumulative risk by age 70y of 65% in *BRCA1*-mutation carriers and up to 45% in *BRCA2*-mutation carriers [16], compared with a lifetime risk of 13% in average risk women. *BRCA1*-deficient and *BRCA2*-deficient tumors have distinct T cell and myeloid populations that impact response to immunotherapy [17], and low expression of BRCA1 protein in breast tumors is associated with increased CD8^+^ T cell infiltration, higher tumor proliferation, and poor survival [18]. Considering higher CD8^+^ T cell counts within breast tumors are generally associated with more favorable survival outcomes [19], these studies suggest that susceptible high risk mammary microenvironments have unique immune landscapes that may be present even before tumor initiation. Compared to pathologically normal breast tissue, benign breast disease (BBD) showed increased densities of dendritic cells, macrophages, CD8^+^ T cells, and B cells near lobules, but pre-malignant BBD that later developed into breast cancer had lower B cell density compared to BBD that did not develop into breast cancer [20]. These findings further support the concept that unique immune milieus likely develop in breast tissues over time to influence breast cancer susceptibility as well as progression. Here, we examined changes in immune cell densities in pathologically normal breast tissue as a function of age from samples that consist of both genetically average risk and genetically high risk women.

T cells, B cells, and macrophages were quantified in non-cancer breast tissue from prophylactic mastectomies. T cells and macrophages were abundant within and nearby normal epithelial ducts and alveoli, emphasizing the likelihood of impactful immune-epithelial interactions. Intralobular stromal and peri-epithelial immune cell densities showed that CD3^+^ T cell and CD20^+^ B cell densities decreased with age and that this decrease may happen more rapidly in genetically high risk tissue compared to genetically average risk tissue. Whereas densities of CD163^−^ M1 macrophages were similar in peri-epithelial and intralobular stroma regions, CD163^+^ M2-polarized macrophages had higher densities in the intralobular stroma compared to peri-epithelium in both young and old tissues. The age-dependent changes and localization differences in immune cell densities observed in situ are consistent with reduced peri-epithelial immune surveillance and suggestive of increased immunosuppression in the intralobular stromal microenvironment.

## Methods

### Participants, Samples, and Donor Demographics

Donors (24–74 years of age) at City of Hope (COH) consented to donate discarded breast tissue and/or blood (IRB no. 17185 and 15,418). Only normal tissues that were histologically confirmed to be non-cancerous were used in the study. Tissues were classified as either peripheral-to-tumor (P), contralateral-to-tumor (C), or prophylactic mastectomy (PM). For this study, women with germline variants that are understood to substantially increase risk of BC (e.g., mutations in BRCA1 or BRCA2) were considered genetically high risk (HR), and those with the absence of germline variants were considered genetically average risk (AR). Donors underwent surgical mastectomy and discarded breast tissues were examined by the Pathology Core and verified to be tumor free. Donor metadata (e.g., age, BMI, risk status) were collected, de-identified, and used in statistical analyses.

### Immune Cell Isolation from Breast Tissues

Fresh surgical discarded tissues were processed and frozen following previously described methods [21]. Filtrate pool ampoules (filtrates) from this process containing mesenchymal, epithelial, and immune cells, and small pieces of vasculature were selected from each specimen for analyses of immune cells originating from breast tissue.

### Flow Cytometry of Filtrates

Filtrate ampoules were removed from liquid nitrogen, thawed in a 37 °C water bath, added to 4 mL of media (RPMI + 10% FBS) in 15 mL conical tubes, and gently resuspended. Samples were incubated at 37 °C for 3 h to allow recovery of surface marker expression, and the suspensions were passed through 45um filters. Cells were centrifuged, and pellets were resuspended in antibody solution. Cells were stained with anti-CD45-APC (BioLegend, Cat. 368512), anti-CD3-FITC (BioLegend, Cat. 300406), anti-CD19-PE (BioLegend, Cat. 302208), and anti-CD14-PerCP/Cy5.5 (BioLegend, Cat. 367110) to identify leukocytes, T cells, B cells, and monocytes/macrophages, respectively. The marker CD14 detects monocytes and macrophages and was used in flow cytometry of filtrates rather than CD68 to allow a more direct comparison to CD14^+^ monocytes detected in donor-matched peripheral blood. The presence of other leukocyte subsets in the filtrates was not accounted for (e.g., NK cells and granulocytes). Gating for each cell type in the flow cytometry analysis was performed in a way that eliminated any ambiguously positive results, as such, the percentage of the three cell types did not add up to 100% for a given donor. Viability control stain 7-AAD was used to exclude dead cells from the analysis. Stained cells were analyzed on a BD Accuri™ C6 flow cytometer, and FlowJo was used for color compensation and gating.

### Multiplex Immunohistochemistry and Slide Imaging

Surgically discarded breast tissue was formalin-fixed and paraffin-embedded (FFPE) at the COH Solid Tumor Pathology Core. FFPE tissues were sectioned at a thickness of 5 μm and adhered to positively charged glass slides.

FFPE sections were triple-stained via immunohistochemistry (IHC) protocol for T cells using anti-CD3 primary antibody (clone SGV6, rabbit monoclonal), for B cells using anti-CD20 (clone L26, mouse monoclonal), and for macrophages using anti-CD68 (clone KP-1, rabbit monoclonal). Staining was performed on Ventana Discovery Ultra Automated IHC Stainer (Ventana Medical Systems, Roche Diagnostics, Indianapolis, USA). First, the slides were loaded onto the instrument, and deparaffinization, rehydration, endogenous peroxidase activity inhibition, and antigen retrieval at pH 8.5 for 64 min were performed. Then, the three antigens were sequentially detected by addition of primary antibodies; heat inactivation was used to prevent antibody cross-reactivity between the same species. Following each primary antibody incubation, DISCOVERY anti-Rabbit HQ or NP, DISCOVERY anti-Mouse HQ or NP, and DISCOVERY anti-HQ-HRP or anti-NP-AP were incubated. The stains were then visualized with DISCOVERY Purple Kit, DISCOVERY Teal Kit, and DISCOVERY Yellow Kit, respectively, counterstained with hematoxylin, and cover-slipped.

The same IHC process was followed for double-staining of pro-inflammatory type (M1) and anti-inflammatory type (M2) macrophages, except using the primary antibodies anti-CD68 (clone KP-1, mouse monoclonal) and anti-CD163 (clone MRQ-26, mouse monoclonal). First, anti-CD163 was added, followed by incubation with DISCOVERY anti-mouse HQ and DISCOVERY anti-HQ-HRP, and visualized by DISCOVERY Teal-HRP kit. Then, after heat inactivation, anti-CD68 was added, followed by incubation with DISCOVERY anti-Mouse NP and DISCOVERY anti-NP-AP, visualized by DISCOVERY Yellow-AP kit, counterstained with hematoxylin, and cover-slipped. Hematoxylin and all IHC antibodies were from Ventana.

Some of the yellow CD68 staining, which appeared as speckles not associated with nuclear counterstain, was initially interpreted as non-specific staining thought to be due to the use of multiple chromogens in the multiplex IHC experiments. However, after consultation with a pathologist and repeating IHC staining of adjacent tissue sections with the traditional chromogen 3,3′-Diaminobenzidine (DAB) and anti-CD68 antibody, which yielded identical staining results, it was determined to be true CD68 staining (Fig. S1). Further consideration led to the conclusion that the yellow speckles were CD68 protein on macrophages that were sliced in the tissue, leaving only fragments of the types of large macrophages that survey the epithelium through dendritic cell-like movement [22]. For consistency between specimens, a threshold was set in the cell classification algorithm to only count as macrophages the areas of yellow CD68 staining with an associated nucleus.

To enable use in image analysis, stained tissue sections were digitized using the VENTANA iScan HT whole slide scanner (Roche) at 20x objective, with a scan resolution of 0.465 μm per pixel.

### Automated Image Analysis to Quantify Triple and Double-Stained IHC Tissues

Machine-learning algorithms for image analysis were customized in the VisioPharm software (Denmark, 2019.11), as detailed below, to segment tissue and classify cells from triple-stained slides with markers for CD3, CD20, and CD68, and double-stained slides with markers for CD68 and CD163.

T cells, B cells, and macrophages were quantified from digitized images of breast tissue sections from 102 donors. CD3^+^/CD20^−^/CD68^−^ (purple-stained cells) were counted as T cells, CD3^−^/CD20^+^/CD68^−^ (teal-stained cells) were counted as B cells, and CD3^−^/CD20^−^/CD68^+^ (yellow-stained cells) were counted as macrophages.

M1 and M2 macrophages were quantified from tissue images from 17 donors (eight ≤ 41y and nine ≥ 58y). CD68^+^/CD163^−^ (yellow-stained cells) were counted as M1, and CD163^+^ (CD163^+^/CD68^−^ and CD163^+^/CD68^+^ double-positive, teal- and green-stained cells, respectively) were counted as M2.

Scientists were blinded to age of donors until after automated cell quantification from images was complete. The objective quantification of immune cells required the following three major steps. First, tissue was segmented to isolate epithelium-enriched regions from fat and stroma. Second, epithelium-enriched regions were further segmented to distinguish epithelial cells of ducts and lobules (peri-epithelium) from intralobular stroma. Third, quantification of individual immune cells within epithelium-enriched regions and classification of each cell type as either directly localized to peri-epithelium, or within intralobular stroma and localized away from epithelium. Detailed description of the three steps follows.

### Segmentation of Epithelium-Enriched Regions from Fat and Stroma

We utilized a deep learning model in the image analysis software VisioPharm to segment and classify the different tissue compartments. In this study, we used the u-net-based deep learning model that is based on the convolutional neural network and we utilized the network architecture [23]. The ReLU activation used is of the form f (x) = max (0, x). An input image size of 512*512 was used for training the model. Using IHC whole slides as reference images, the epithelium-enriched regions and fat and stroma regions were annotated by the pathologist. The training regions were annotated within the VisioPharm framework and act as a ground truth for training the model. Data augmentation was performed during the training process due to small sample size. A total of 10 whole slide images of 35,000*28,000 pixel size were used for training the algorithm with two different classifiers: epithelium-enriched regions (EER, blue) and fat or stroma (FSR, green) shown in Fig. S2A. Multiple training regions were annotated for each classifier in the samples. Training samples contained images with diverse staining patterns and different staining intensities to address tissue heterogeneity. A total of 45 annotations were made on different training samples and the model was trained on these images. Once the model was trained, it was tested on a large cohort of samples (n=100) and annotations were made in EER only.

### Segmentation of Epithelial Cells of Ducts and Lobules from Intralobular Stroma

In this step, we only consider the region annotated by step 1 for further segmentation of the EER region. Instead of directly segmenting the intralobular epithelial region this two-stage approach was adopted to increase the segmentation accuracy. We used the u-net architecture described above to train a two-class classifier to segment the EER into peri-epithelium (PE, blue) and intralobular stroma (ILS, green). The training images and labels were generated as shown in Fig. S2B. The training regions were annotated within the VisioPharm framework and act as a ground truth for training the model. The u-net was trained for 6000 epochs until the validation error was less than 10 percent. The trained model was saved and tested on a large cohort of samples (n=100) and annotations were made both on PE and ILS to estimate the immune cell count within the respective regions.

### Quantification of Individual Immune Cells

**Fig. 1:**
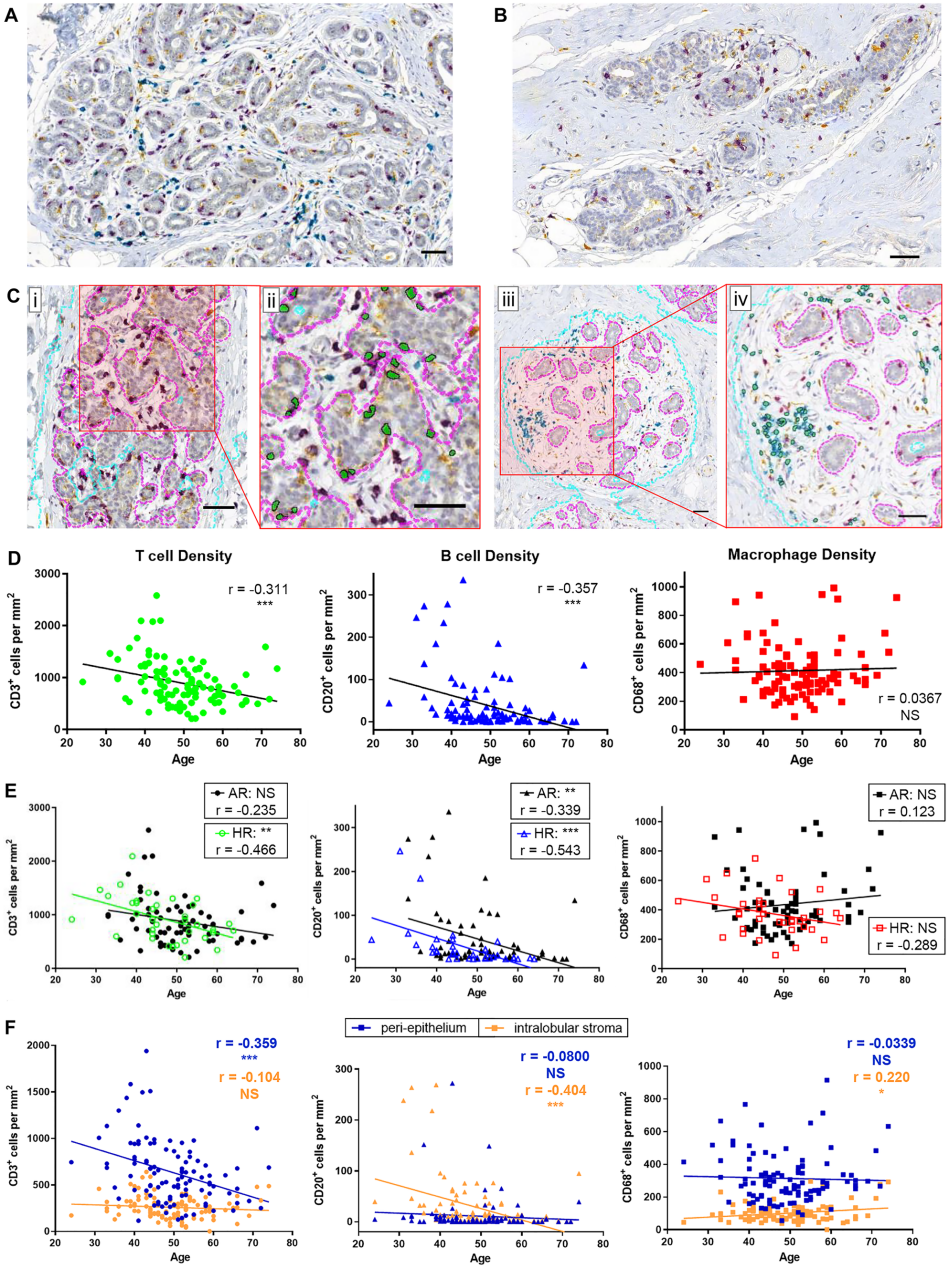
Immune cells closely associated with mammary epithelium and their density in situ changed with age. Representative images of IHC triple-stained pathologically normal breast tissue from **A** young (33y) and **B** older (66y) donors. Purple (CD3), Teal (CD20), Yellow (CD68). Scalebars are 50 µm. **C** Machine-learning algorithms were used to first segment the tissue into fat and stroma regions (FSR) or epithelium-enriched regions (EER) (light blue dotted lines), and then to further segment tissue within EER as intralobular stroma (ILS) or peri-epithelium (PE) (magenta dotted lines). The three immune cell types were then classified within EER as being either within the PE (within magenta dotted lines) or within the ILS (within light blue and outside of magenta dotted lines) and quantified in each area. (i) and (iii) show segmentation output only, (ii) shows cell classification output of CD3^+^ T cells within PE from inset in (i), and (iv) shows cell classification output of CD20^+^ B cells within ILS from inset in (iii). Scalebars are 50 µm. **D** Total density (cells per mm^2^) of each immune cell type in EER was quantified in situ and compared with donor age by linear regression (n = 102). r, correlation coefficient, and p-value of each regression are indicated. **E** Age regressions of each immune cell type when samples are grouped into AR (filled shapes; n = 67) or HR (open shapes; n = 36). r and p-value of each regression are indicated. **F** Immune cell densities (cells per mm^2^) quantified in PE (blue) and in ILS and distant from the epithelium (orange) (n = 102). r and p-value of each regression are indicated. * p < 0.05, ** p < 0.01, *** p < 0.001, NS = not significant (P > 0.05)

Within the VisioPharm framework, we utilized the color deconvolution algorithm along with the binary image operation to segment and count the individual immune cells. Using color deconvolution, individual immune cells stained for CD3 (purple), CD20 (teal), and CD68 (yellow) were deconvolved from the hematoxylin counterstain. The deconvolved positive stain channel was further used for the cell segmentation. In this process the deconvolved positive stain channel was smoothed using the mean filter and Poly Blob filter to segment the individual cells. The Poly Blob filter size was manually estimated to be 33µm. Binary objects less than 15 µm were filtered out and cells of image intensity less than 50 were considered positive cells for each immune marker. Image analysis parameters such as cell count and area of peri-epithelium and intralobular stroma were estimated. The ratio of cell count to area of the intralobular compartments estimate the cell density within their corresponding compartments (cells/mm^2^). Figure 1C shows the segmentation and classification output of CD3 (Fig. 1Ci-ii) and CD20 (Fig. 1Ciii-iv) in the peri-epithelium and intralobular stroma, respectively.

### PBMC Isolation from Whole Blood

Peripheral blood mononuclear cells (PBMCs) were isolated from whole blood of 20 donors, from which breast tissue was obtained on the same day, using Lymphoprep™ density gradient medium and SepMate™ PBMC isolation tubes (StemCell). PBMCs were stained with antibodies for CD3, CD19, CD14, and CD45 and analyzed via flow cytometry in the same manner as filtrates.

### In Silico Transcriptional Signature Analyses

As described in the original article (NCBI GEO GSE102088 [24]), frozen breast tissue obtained from reduction mammoplasty (RM) of 121 healthy women underwent RNA extraction and was profiled via the GeneChip® Human Transcriptome Array 2.0 (Affymetrix Inc, Santa Clara, CA). The data were log_2_-transformed and quantile-normalized by the original authors. All further analyses for this study were conducted in R. Published immune gene signatures [25–28] were aggregated into a single dataset for further downstream analysis. These immune signatures give an estimate of the proportions or enrichment of immune cell types as well as of immunomodulatory signaling (e.g., IFN and TGF-β response) present in bulk tissue, allowing the comparison of many immune cell subsets across a range of ages. The samples were grouped by age, with ≤ 35 years old defined as young (n = 51) and ≥ 50 years old defined as older (n = 23), and gene annotations were updated. Identified gene probes with maximum average gene expression across all samples were filtered for and mean center scaled prior to analyses. The Wilcoxon rank-sum test was used for statistical analysis between young and older age groups. The Holm-Bonferroni method was used to correct for multiple testing across all signatures and calculate adjusted p-values (adj. p-val). These analyses were performed to determine age-dependent immune changes in an orthogonal dataset from normal breast tissue with no previous or current BC and no known genetic mutations.

### Statistical Analysis

GraphPad Prism 8 was used for data visualization and statistical analyses of all data except the in silico analyses, which used the statistical software R. All boxplots used the Tukey method for plotting whiskers and outliers. The relationships between variables were analyzed by simple linear regression, Pearson’s correlation, two-tailed t-tests, or ordinary one-way analysis of variance (ANOVA). Paired t-tests were used to compare PE to ILS data from the same individuals and unpaired t-tests were used to compare AR to HR data between different individuals. Any significant ANOVA was followed by post-hoc analyses using Tukey’s multiple comparisons test, corrected for multiple comparisons using statistical hypothesis testing, and reported as adj. p-val. For all statistical analyses, a two-sided p-value less than 0.05 was considered significant and the following symbols were used to indicate significance: * p ≤ 0.05, ** p ≤ 0.01, *** p ≤ 0.001, **** p ≤ 0.0001, NS (p-value is not significant).

## Results

### Density of T Cells, B Cells, and Macrophages by In Situ IHC Analysis

To determine how immune milieus change with age in breast tissue, we examined densities of common immune cell types in pathologically normal breast tissue. Multiplex IHC of FFPE sections for single marker identification of T cells (CD3), B cells (CD20), and macrophages (CD68) demonstrated that immune cells closely associated with epithelium-enriched regions (EER) in normal breast tissue (Fig. 1A-B). Manual examination showed most of the immune cell density was localized in EER, rather than in adipo-stromal regions, and T cells and macrophages were visible within the epithelial bilayers. Thus, cell counting algorithms were optimized for detecting immune cells in EER (Fig. 1C and Fig. S2) and quantified per mm^2^ to account for variation in total area of EER regions between donors.

T cells were the most abundant immune cell type in EER, followed by macrophages, then B cells. T cell and B cell densities decreased in breast tissue with age (p = 0.001 and p < 0.001, respectively), whereas macrophage density did not change significantly with age (p = 0.713) when considering all samples irrespective of genetic risk status (Fig. 1D). When samples were separated by genetic risk status, T cell and B cell density decline with age was more prominent in HR than in AR, and opposite directions of regression lines suggested macrophage density may trend downward with age in HR and upward with age in AR (Fig. 1E), but neither was statistically significant. Thus, the germline mutations associated with HR status may have differentially impacted the immune milieu of the mammary glands. One-way analysis of variance (ANOVA) indicated that variations in T cell and B cell densities in HR tissues (p = 0.006 and p = 0.013, respectively) and B cell and macrophage densities in AR tissues (p < 0.001 and p = 0.001, respectively) may be explained by age range (Fig. S3A). No significant differences in densities of the three immune cell types were seen when samples were grouped by other factors that could influence immune milieus, including: body mass index (BMI), tissue type, or receptor subtype of peripheral or contralateral tumor tissue (Fig. S4A-C). Parity did not show a strong effect on immune composition, as only B cells showed a potentially significant association that was not robust to outliers (Fig. S4D). T cell and B cell densities were lower in the post-menopausal group compared to the pre-menopausal group, but there were only three women in our dataset who were post-menopausal and < 50y so it was not possible to distinguish from the effect of age (Fig. S4E).

The densities of T cells and macrophages in peri-epithelial regions were greater than the densities of these two immune cell types in the intralobular stroma (p < 0.001 between PE and ILS for both T cells and macrophages, paired t-tests) (Fig. 1F and Fig. S3B, left and right panels), whereas B cells were more abundant in the intralobular stroma than in the peri-epithelium (p = 0.005, paired t-test) (Fig. 1F and Fig. S3B, center panel). Mean density of intralobular stromal T cells was 259 ± 123 cells/mm^2^ in all samples, whereas mean density of peri-epithelial T cells was 849 ± 357 cells/mm^2^ in women ≤ 41y (adj. p < 0.001 between ILS T cells of all and PE T cells of ≤ 41y) and 477 ± 222 cells/mm^2^ in women ≥ 58y (adj. p = 0.006 between ILS T cells of all and PE T cells of ≥ 58y) (Fig. S3C). Mean density of intralobular stromal macrophages was 101 ± 54 cells/mm^2^, whereas mean density of peri-epithelial macrophages was 313 ± 152 cells/mm^2^ (two-tailed, paired t-test p < 0.001) (Fig. S3B). Mean density of peri-epithelial B cells was 11 ± 35 cells/mm^2^ in all samples, whereas mean density of intralobular stromal B cells was 75 ± 92 cells/mm^2^ in women ≤ 41y (adj. p < 0.001 between PE B cells of all and ILS B cells of ≤ 41y) and dropped to 9 ± 22 cells/mm^2^ in women ≥ 58y (adj. p = 0.998 between PE B cells of all and ILS B cells of ≥ 58y) (Fig. S3C). This shows that B cells were more abundant in the intralobular stroma than in the peri-epithelium of young women, but their abundance in the intralobular stroma was lost in older women. The mean density of peri-epithelial T cells decreased by nearly fifty percent with age and approached the density of intralobular stromal T cells (Fig. 1F and Fig. S3C, left panel). This decline in peri-epithelial T cell density may indicate reduced T cell surveillance of the epithelium with age.

### Proportions of Peri-Epithelial Immune Subsets by Flow Cytometry Analysis

**Fig. 2:**
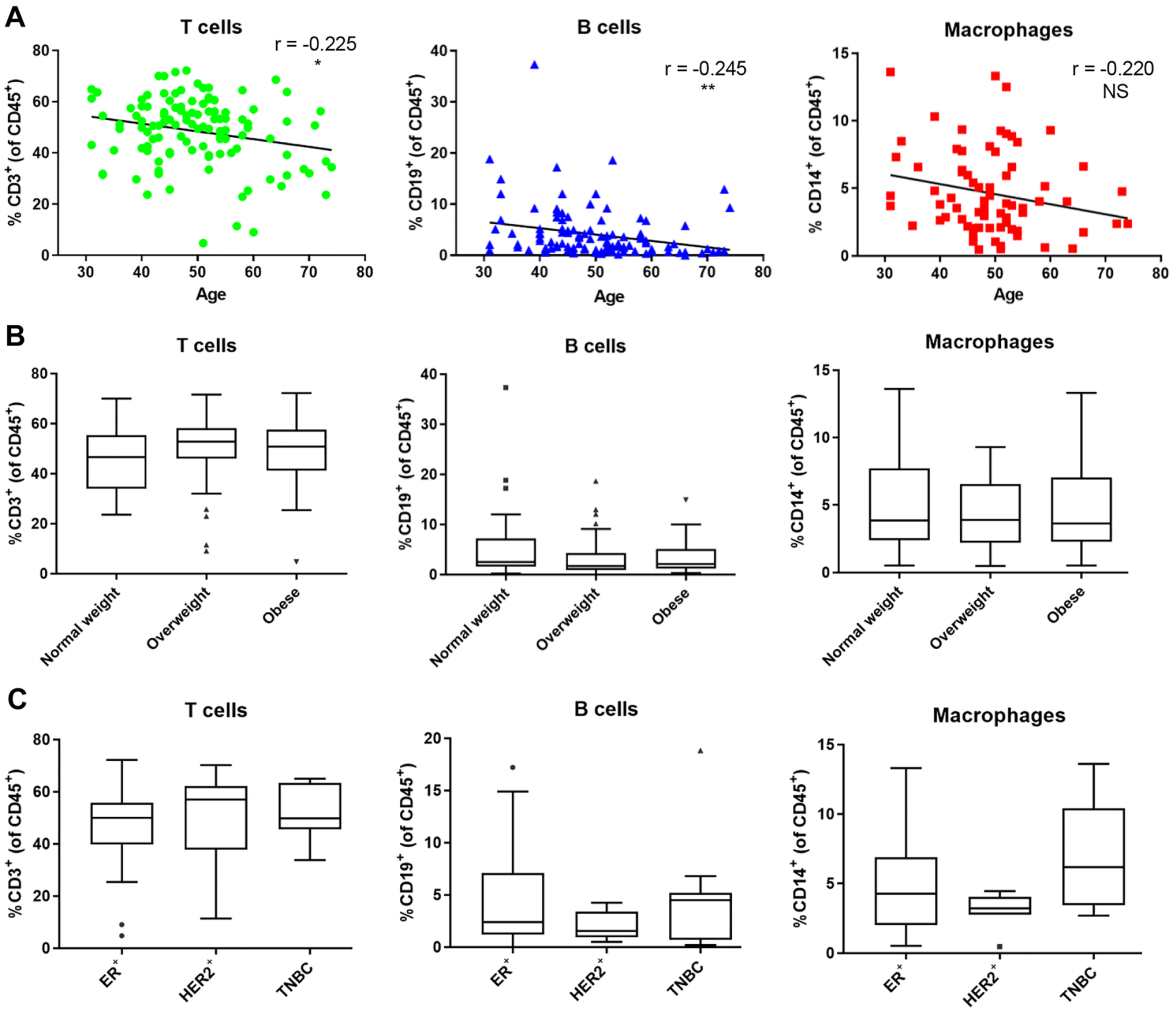
Mammary gland peri-epithelial immune cells were isolated and measured by flow cytometry. **A** Immune cell proportions quantified via flow cytometry and compared with donor age by linear regression. T cells (CD3^+^) (n = 116), B cells (CD19^+^) (n = 116), Macrophages (CD14^+^) (n = 74). r and p-value of each regression are indicated. **B** ANOVA for immune cell type and BMI (n = 115): normal weight (18.5 ≥ BMI < 25) (n = 37), overweight (25 ≥ BMI < 30) (n = 43), obese (BMI ≥ 30) (n = 35). **C** ANOVA for immune cell type and tumor receptor status in contralateral tumor tissue (n = 78): ER^+^ (n = 53), HER2^+^ (n = 14), TNBC (n = 11). All ANOVA p-values were NS

The fraction of cells that remained following isolation of mammary epithelial organoids from processed breast tissue specimens, the filtrate fraction, was enriched for immune cells of the peri-epithelial region of mammary gland [21]. At least 10,000 viable mononuclear events per filtrate sample were analyzed by flow cytometry (n = 116). CD45^+^ leukocytes detected in the filtrates comprised 11 ± 7% of mononuclear events in filtrates, the remainder of nucleated cells consisted of fibroblasts, epithelial cells, and endothelial cells. Quantification of immune cells from the CD45^+^ portion of filtrates showed proportions of T cells and B cells, identified by CD3^+^ and CD19^+^, respectively, decreased with age (p = 0.015 and p = 0.008, respectively) (Fig. 2A). Macrophage proportions identified by CD14^+^ expression showed no significant change with age (p = 0.060). Thus, using a second analytic modality in a parallel set of samples, we observed similar patterns of change with age compared to the in situ analyses of immune milieus in EER of mammary gland.

High body mass index (BMI) is a common risk factor for many health issues, including postmenopausal BC, and obesity (BMI ≥ 30) is associated with altered immune response, including increased chronic inflammation, however, variation in these immune subsets was not explained by BMI (Fig. 2B). Focusing on the filtrate fractions from contralateral mastectomies, we detected no significant difference in proportions of immune cell subsets associated with receptor status (i.e., ER^+^, HER2^+^, or TNBC) of the tumor in the affected breast (Fig. 2C). Unlike immune milieus inside tumor tissues that are dependent on tumor subtype [29], these data suggested immune milieus of the contralateral normal tissue were primarily correlated with age.

Because in situ analyses showed T cells and macrophages within the epithelial bilayer, we questioned whether immune cells could be a commonly overlooked cellular component of standard epithelial organoid preparations or even pre-stasis human mammary epithelial cells cultured from epithelial organoids. We examined the different fractions generated during a mammary epithelial organoid preparation for cells expressing CD45 using flow cytometry. The fractions examined were: filtrates, uncultured organoids, and cultured pre-stasis epithelial cells at passage four and five [21]. The majority of CD45^+^ immune cells were in the filtrate fractions, whereas there were few percent of total CD45^+^ cells detected in digested organoid fractions, and none in the cultured epithelial cells (Fig. S5).

### Skewed Intralobular M2 Macrophage Polarization

**Fig. 3:**
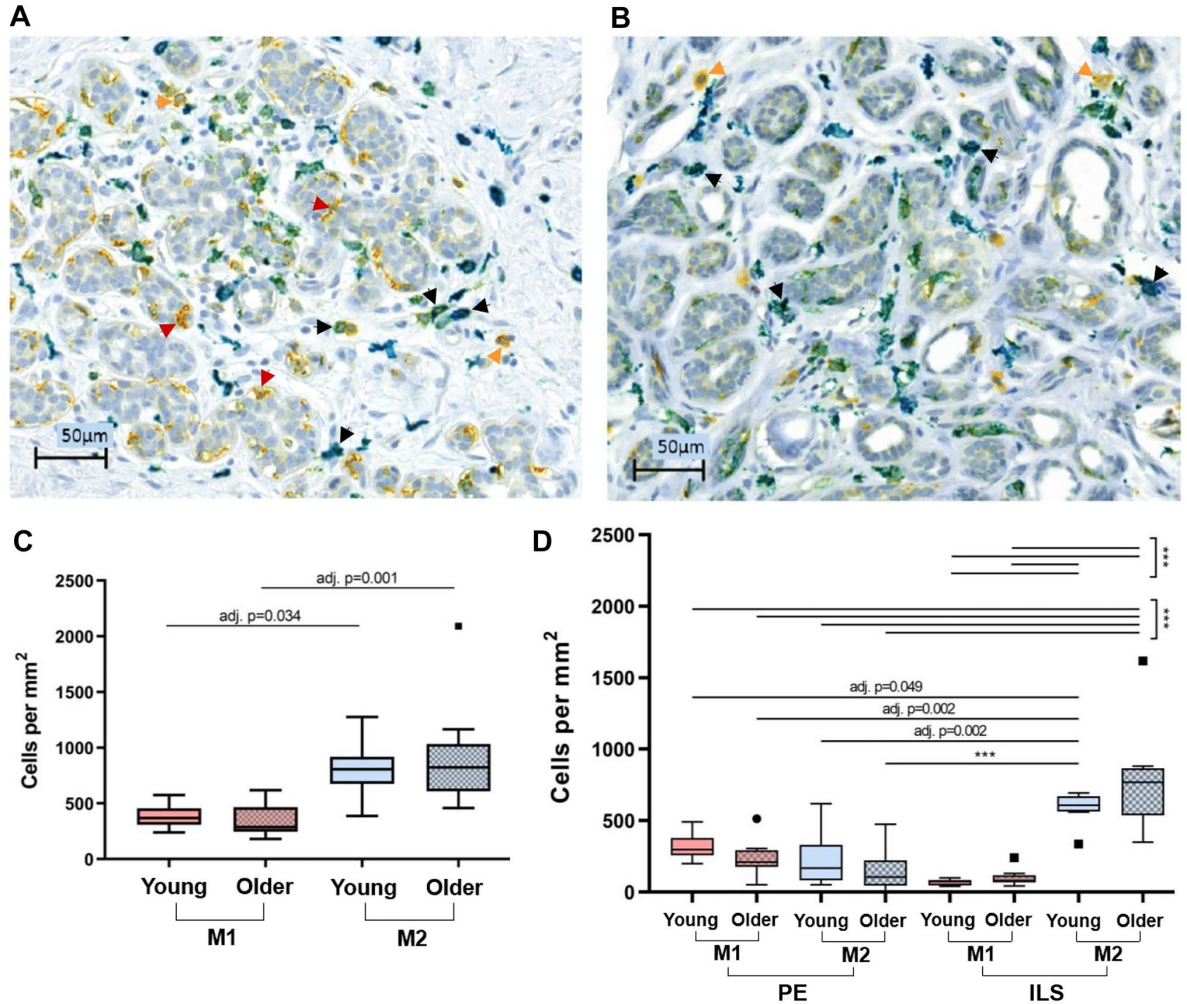
M1 and M2 macrophage quantification in IHC-stained breast tissue further characterized the aging immune phenotype. Representative images of IHC double-stained breast tissue from **A** young and **B** older donors. Yellow (CD68^+^), Teal (CD163^+^), Green (CD163^+^/CD68^+^). M1 (CD68^+^/CD163^−^) and M2 (CD163^+^/CD68^−^ and CD163^+^/CD68^+^). Red arrowheads point to peri-epithelial (PE) M1 macrophages, orange arrowheads point to intralobular stromal (ILS) M1 macrophages, and black arrowheads point to ILS M2 macrophages. Scalebars are 50 µm. **C** Density of M1 and M2 macrophages quantified in situ from young (≤ 41y, n = 8) and older (≥ 58y, n = 9) donor age groups. **D** Density of M1 and M2 macrophages in PE or ILS in each age group. Adjusted p-values (adj. p-val) of ANOVA post-hoc analyses between groups are indicated

Pro-inflammatory M1 macrophages and anti-inflammatory M2 macrophages were quantified in EER from a subset of younger (≤ 41y, n = 8) and older (≥ 58y, n = 9) specimens to determine if there were differences in macrophage polarization with age or tissue location. M1 macrophages (CD68^+^/CD163^−^) are stained yellow, and M2 macrophages (CD163^+^) are teal (CD163^+^/CD68^−^) and green (CD163^+^/CD68^+^ double-positive (DP)) in IHC-stained breast tissues (Fig. 3A-B). Comparison of both CD163^+^ populations (CD163^+^/CD68^−^ and CD163^+^/CD68^+^) in young and older tissue groups showed no significant change in density of either population with age (Fig. S6). M2 density was higher than M1 density in both young (adj. p = 0.034) and older tissues (adj. p = 0.001), and though decrease in M1 and increase in M2 with age were not statistically significant, the difference between mean M1 and mean M2 density increased in older tissue (from 423.3 to 582.8 cells per mm^2^), suggesting a slight shift in macrophage subtype favoring M2 polarization (Fig. 3C). When examined according to location of macrophages in either peri-epithelial or intralobular stroma regions, it became clear that the greater M2 density compared to M1 density is due to high M2 density in intralobular stroma. Density of M2 macrophages in intralobular stroma of both young and older tissues is significantly greater than density of all other M1 and M2 populations in peri-epithelium and intralobular stroma (Fig. 3D). The increased M2 macrophage density in intralobular stroma suggests increased immunosuppression in the mammary gland microenvironment.

### Comparison of Immune Milieus in Matched Breast Tissue and Peripheral Blood

**Fig. 4:**
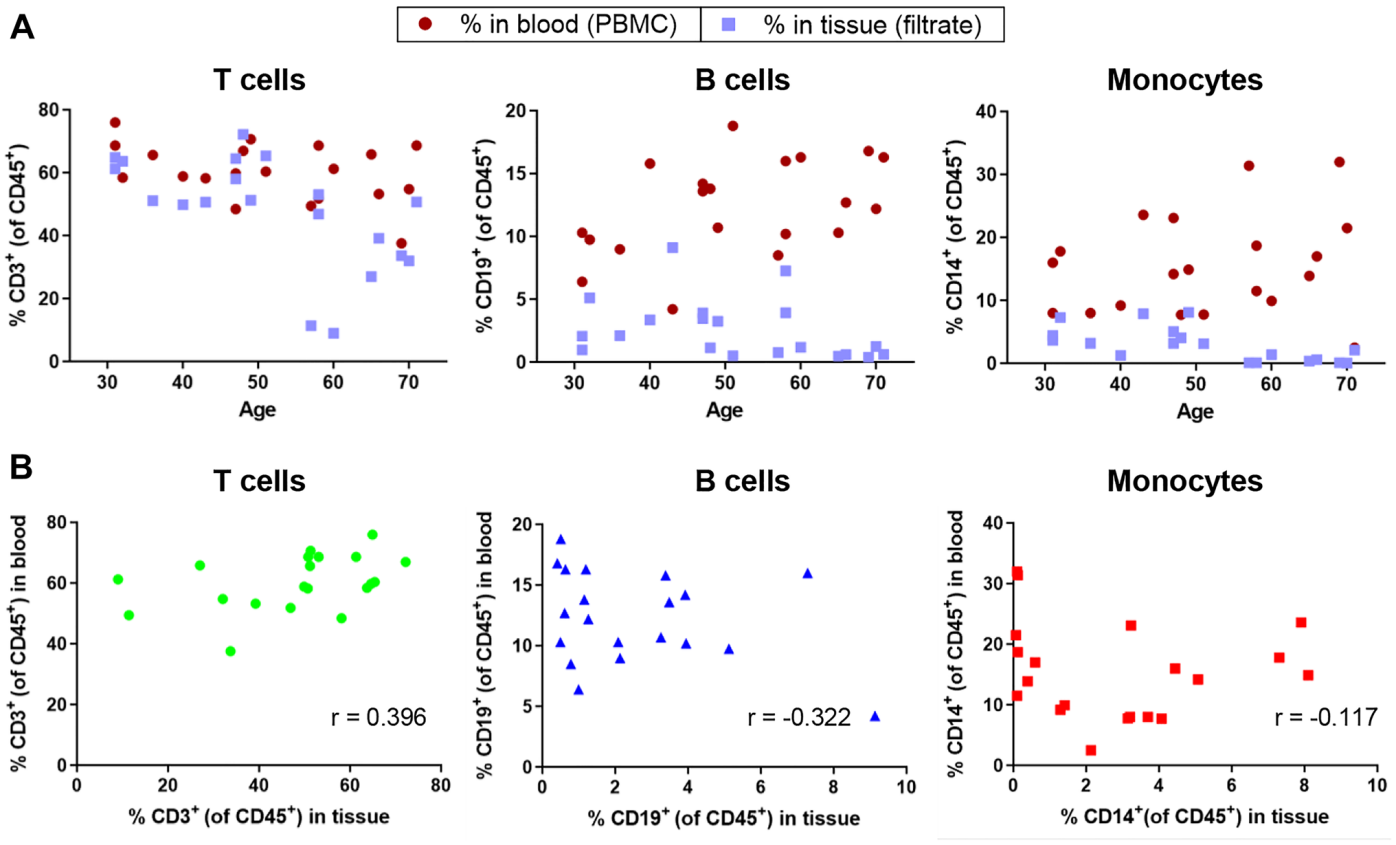
Immune cell proportions were not correlated in donor-matched peripheral blood and breast tissue. Donor-matched PBMCs (n = 20) were analyzed by flow cytometry in the same manner as filtrates to determine proportions of immune cells. **A** Percent of each immune cell type in blood (red circles) and in tissue (blue squares) compared with donor age. **B** Pearson’s correlation analysis for each cell type in blood and tissue. r, Pearson’s correlation coefficient, is indicated. P-values were all NS

Peripheral blood is often examined as a proxy for immune cell composition in solid tissues. We determined whether immune cell proportions in filtrate fractions from mammary gland preparations were reflected in the peripheral blood mononuclear cells (PBMCs). Matched breast tissue and PBMCs were examined from n = 20 individuals. Proportions of T cells, B cells, and monocytes in tissue and blood did not exhibit similar patterns of change with age (Fig. 4A). In blood, T cell proportions showed a downward trend and monocyte proportions showed an upward trend with age. Proportions of immune cells in peri-epithelial regions and in matched peripheral blood were not correlated (Fig. 4B). Therefore, PBMCs are not likely to be a suitable proxy for studying age-dependent changes in breast tissue immune milieus.

### Immune Cell Transcriptional Signatures of Reduction Mammoplasty Samples

We examined publicly available gene expression data from bulk tissue reduction mammoplasty (RM) samples for the presence of immune signatures [25–28]. Older breast tissue exhibited higher signature scores for innate immune cells associated with inflammation and immunosuppression, including macrophages (adj. p = 0.013), mast cells (adj. p = 0.014), neutrophils (adj. p = 3.20E-10 and adj. p = 6.70E-07), and NK CD56_dim_ cells (adj. p = 0.0022) (Fig. S7, Table S1). Older RM had signature scores consistent with decreased function of adaptive immunity and recognition, including decreased B cells (adj. p = 0.0027), T helper cells (adj. p = 1.10E-11), and central memory T cells (Tcm) (adj. p = 4.00E-06) (Fig. S7, Table S1). Additionally, older breast tissue exhibited higher signature scores for angiogenesis (adj. p = 1.40E-13), which involves both innate and adaptive immune functions. This combination of generally increased innate immune cell and decreased adaptive immune cell signatures in a dataset that was orthogonal to our own samples was consistent with the age-dependent changes observed in the immune subsets we quantified in situ.

## Discussion

The immune system is increasingly appreciated as a determinant of BC progression. Aging is the greatest risk factor for BC, and we performed a survey of immune cell subsets in normal breast tissue as a function of age to better understand whether there were changes that could be related to breast cancer susceptibility. We examined immune milieus in peri-epithelial and intralobular stroma regions of histologically normal breast tissue to better understand whether there were age-dependent changes. T cells and macrophages were intimately associated within the bilayers of epithelial cells; indeed, without proper biomarkers they could have been mistaken for epithelial cells in tissue sections. Chronic sterile inflammation, so called “inflammaging”, has been proposed to be a driving factor of many diseases of aging including cancer. Our in situ macrophage results showing no significant increase in M1 macrophage density with age did not support the concept that an increased inflammatory tissue microenvironment emerges in breast with age. However, the density of anti-inflammatory M2 macrophages in the intralobular stroma was greater than pro-inflammatory M1 macrophages, and this difference between M2 and M1 density increased with age, raising the possibility that age-related BC susceptibility is aided by increased immunosuppression in the microenvironment. Meanwhile, the adaptive immune milieu changed significantly, in that both T cells and B cells decreased in density with age in two parallel sample sets. In addition, decreased transcriptional signatures with age for B cells and several T cell subsets were detected in a third dataset. Overall, these changes in immune milieus in breast with age are consistent with the immunosenescence concept, which posits that decreased function of adaptive immunity with age is a driver of susceptibility to cancer and other age-related maladies [30].

The density of peri-epithelial T cells decreases with age, suggesting a reduction in T cell-to-epithelial cell communication or even a decline in direct surveillance of the epithelium in aged breast tissue. Reduction in T cell migration to the epithelium for immune surveillance could contribute to tumor development [31]. A previous study of normal breast tissues with or without lobulitis showed the most abundant type of T cells in breast were CD8^+^ cytotoxic T cells [32]. Assuming a certain degree of consistency across normal tissues, the age-dependent decline of T cell-to-epithelial cell interactions observed in our specimens would be consistent with reduced immune surveillance by cytotoxic T cells.

Mouse studies have shown that immune cell subsets are involved in sculpting mammary gland development. Cells of the innate immune system (e.g., macrophages, eosinophils, and mast cells) were shown to promote postnatal mammary gland development by increasing branching and growth [10–12]. Conversely, the adaptive immune system was shown to suppress postnatal mammary development by inhibiting luminal epithelial differentiation through IFN-ɣ released from Th1 cells that interact with mammary-resident dendritic (CD11c^+^) cells [13]. It is important to consider potential effects of immune cells on the luminal epithelial cells in the aging and cancer context [33] because mature luminal cells are the chief suspects for the cancer cells-of-origin of the luminal subtype cancers that are strongly associated with age [34]. We previously showed that two of the most striking age-dependent changes in human mammary epithelia are the accumulation of mature luminal cells and progenitor cells with a basal differentiation bias [5, 7]. The capacity of CD4^+^ T cells to differentiate into Th1 effector cells tends to decrease with age [35], thus it is tempting to speculate that a subsequent decrease in IFN-ɣ production in the microenvironment of the mammary gland lifts the inhibition on luminal differentiation enabling accumulation of these putative cancer cells-of-origin with age.

One study identified changes in immune populations in the aging mouse mammary gland [36]. Many of the findings in that paper are the opposite of ours (e.g., they see more T and B cells with age in the mouse mammary gland whereas we see less). They also identify two macrophage populations, not specifically terming them M1 and M2, but one macrophage population that expresses the M2 marker, CD163, is more abundant in the stroma, which is in alignment with our results of CD163^+^ M2 macrophages having a greater density in the intralobular stroma compared to in the peri-epithelium. However, they showed a decline in the CD163^+^ macrophage subset with age, making the other subset the dominant type in aged tissue, which is not what we observed in human breast. These results are intriguing, but we are hesitant to directly compare our results from a group of over 100 human tissues with these findings from a single inbred mouse strain with only 3–4 mice per group. This mouse study may be useful for detecting changes with age because the mice within each group are so similar to one another, but the changes cannot be directly translated to humans because of the lack of variation in laboratory mouse strains and the many evolutionary differences. Indeed, experimental outcomes in mice vary depending on the mouse strain background, diet, housing, and other environmental factors [37]. This underscores the importance of using human samples to identify changes that occur in humans, especially to understand complex processes like cancer and aging.

M2 macrophages were defined as expressing the M2-specific marker CD163, a hemoglobin scavenger receptor. CD163 is one of the most common and reliable markers for M2 macrophages and tumor-associated macrophages (TAMs) [38, 39], and it is specific for cells of the monocyte/macrophage lineage [39], but we caution that using a single marker as an indicator of M2 polarization and no additional M1 marker may be overly simplistic. Macrophage polarization exists in a spectrum, and there are several markers that are differentially expressed as a macrophage is polarized to either M1 or M2. The functions of macrophages vary along this range of polarization from M1 to M2, and a polarized macrophage can be converted still further depending on microenvironment influence [40]. In double-stained IHC tissues we designated CD163^+^ cells as M2 macrophages even if they appeared to be negative for CD68. Though rare, other studies have demonstrated the presence of CD163^+^ macrophages lacking CD68 expression [38, 41]. Thus, CD163^+^ (apparent CD68^−^) cells were either M2 macrophages with loss of CD68 expression, or M2 macrophages with CD68 expression, but the darker teal chromogen from CD163 expression was masking the lighter yellow chromogen from colocalized CD68 expression [42]. Indeed, comparison to adjacent tissue slices showed substantial overlap of CD68^+^ staining with CD163^+^/CD68^−^ cells in the double-stained tissue, suggesting these CD163^+^ cells expressed CD68. The increased M2 macrophage density in intralobular stroma is consistent with an interpretation that an immunosuppressive microenvironment emerges in breast tissue. M2 macrophages secrete growth factors and cytokines that modify tissue architecture and enable increased tumor cell proliferation and immunosuppression [43]. This suppressive microenvironment could provide opportunities for expansion of transformed epithelial cell types.

There were disparities between the IHC staining of breast tissue and in silico analysis of reduction mammoplasty transcriptional signatures in that IHC results showed no significant change in macrophage density with age, but significant increases with age were detected in macrophage transcriptional signature and other innate immune populations (mast cells, neutrophils, NK cells). This discrepancy is likely due to the differences in tissue samples and methods of analysis. Transcriptional signatures represent a composite of the expression of multiple genes that give an estimate of the abundance of certain cell types, whereas IHC data show a single protein marker that identifies the discrete cell type of interest. A likely reason for the discrepancy between in silico and IHC results is that the sequencing data were from bulk breast tissue, which includes expression of genes from all cell types in the breast, whereas in IHC cell types are identified by a specific protein marker. Indeed, we observed previously that enrichment of inflammation related genes in mammary epithelial cells is a feature of aging [33].

A limitation of this study is that it focuses on the most predominant immune cell subsets in the breast and ignores other less abundant, but perhaps still physiologically important, immune cell subsets. A deeper characterization determining the abundance and function of other immune cell types would provide a clearer picture of how the immune landscape in breast tissue changes with age. Furthermore, it is possible the flow cytometry data of immune populations are slightly altered due to changes in surface molecule expression that occur while processing the tissue under enzymatic conditions at 37 °C. Various enzymatic and mechanical tissue digestion procedures impact accurate detection of surface marker expression by immune cells [44–46]. Though digestion with collagenase, one of the enzymes used in our tissue processing procedure [21], was shown to have minimal impact on molecules expressed at the surface of blood cells [45], the impact of hyaluronidase (the other enzyme used for tissue processing) on surface molecule expression was not determined. This uncertainty is why the in situ IHC results are considered a more reliable representation of the true immune cell proportions in the human breast compared to the flow cytometry analysis of filtrates. With that said, these limitations were addressed, in part, by analyzing age-related changes in an orthogonal dataset (i.e., transcriptional signatures), which reinforced the changes in immune milieus demonstrated by the in situ data and also indicated changes in additional immune subsets not included in the in situ analysis presented here.

A further limitation to our interpretation of the results of immune changes with age is the presence of other factors impacting the immune milieu that were not considered here. Menstrual cycle stage [47], mammographic density [48], and involution following lactation [49, 50] have all been shown to affect the abundance of immune cells and breast cancer risk, but we did not have this information available for the tissue donors used in this study. It is possible these factors influenced the immune cell densities in the breast tissue, particularly in premenopausal women, and confounded our ability to accurately assess the relationship between immune milieu and age. In the genetically high risk group, the different genetic mutations imposing increased risk for breast cancer might account for some of the variability seen in this population. These additional factors could all lead to increased variation between individuals, and with a larger cohort of women and data on these additional factors, some of this variation may be parsed out. Known factors such as BMI and parity did not explain variation in the immune cell densities in our cohort.

## Conclusions

The in situ characterization presented here shows that immune cells are closely associated with epithelial cells in normal breast tissue, the T cell and B cell densities in epithelial-enriched regions are reduced with age, and immunosuppressive M2 macrophage density is higher than pro-inflammatory M1 macrophage density. Many of these immune cell changes are reflected in the in silico analysis of immune signature scores of bulk tissues from normal RM samples. These changes in immune milieus of the mammary gland suggest provocative immune-epithelial interactions that may presage deleterious changes in immune surveillance with age that increase susceptibility to transforming events, and possibly even contribute to some well-described aging phenotypes in the luminal epithelia. Luminal subtype breast tumors represent about 80% of age-related BCs and are infamously considered “immunologically cold”, which presents challenges for immunotherapeutic approaches [51, 52]. It is tempting to speculate that the “cold” immune microenvironment is a vestige of the aging process. Perhaps interventions meant to prevent general age-related immune decline [53] could be used to prevent age-related breast cancers or improve the likelihood of a more immunogenic tumor microenvironment.

## Supplementary Information

Below is the link to the electronic supplementary material.

Supplementary file 1 Figure S1. 3,3’-Diaminobenzidine (DAB) staining confirmed yellow chromogen staining for CD68 via IHC. Adjacent tissue sections incubated with anti-CD68 monoclonal antibody were visualized with either (A) yellow chromogen or (B) traditional brown DAB chromogen. Scalebars are 20µm. (PDF 176 KB)

Supplementary file 2 Figure S2. Pipelines for training the u-net model in VisioPharm. (A) Pipeline for training the u-net model to classify epithelium-enriched regions (EER) and fat or stroma regions (FSR). (B) Pipeline for training the u-net model to classify peri-epithelium (PE) and intralobular stroma (ILS) within the EER. (PDF 126 KB)

Supplementary file 3 Figure S3. Genetic risk status of tissue and proximity to mammary epithelium yields differences in immune cell densities. (A) ANOVA performed on IHC cell density data separated by BC genetic risk and age group showed age range may explain variations in T cell and B cell densities in HR tissues (p=0.006 and p=0.013, respectively) and variations in B cell and macrophage densities in AR tissues (p<0.001 and p=0.001, respectively). Adj. p-val of post-hoc analyses between age groups are indicated. 20-40y (HR: n=10, AR: n=7), 41-50y (HR: n=12, AR: n=27), 51-59y (HR: n=11, AR: n=24), ≥60y (HR: n=3, AR: n=9). (B) Immune cell densities (cells per mm^2﻿^) quantified in PE (blue) were compared to densities quantified in ILS and distant from the epithelium (orange) (n=102). T cells and macrophages had higher densities in PE and B cells had higher density in ILS. Two-tailed, paired t-test p-values are indicated. (C) Comparisons between T cell or B cell densities in PE and ILS in all (24-74y, n=102), young (≤41y, n=21), and older (≥58y, n=19) age groups show a decline in ILS B cell and PE T cell densities with age. Adj. p-val of post-hoc analyses are indicated. (PDF 200 KB)

Supplementary file 4 Figure S4. Tissue samples grouped by other factors that may influence immune milieu and breast cancer risk. (A) ANOVA for immune cell type and BMI (n=102): normal weight (18.5 ≥ BMI < 25) (n=34), overweight (25 ≥ BMI < 30) (n=39), obese (BMI ≥ 30) (n=29). (B) ANOVA for immune cell type and tissue type (n=102): contralateral, C (n=77), peripheral, P (n=15), prophylactic mastectomy, PM (n=11). (C) ANOVA for immune cell type and receptor subtype of peripheral or contralateral tumor tissue (n=82): ER^+﻿^ (n=56), HER2^+﻿^ (n=11) triple-negative breast cancer, TNBC (n=15). (D) ANOVA for immune cell type and parity, defined as the number of pregnancies resulting in live births, (n=102): 0 (n=21), 1 (n=17), 2 (n=31), 3 (n=20), 4 or more (n=13). The only significant difference was seen in B cells (p=0.048) and post-hoc analysis indicated a difference between the parity groups 0 and ≥4 (adj. p=0.046). (E) Two-tailed, unpaired t-tests were used to compare immune cell densities between samples based on donor menopause status (n=93): pre (n=54), post (n=39). T cell and B cell densities decreased in post-menopause tissues (p=0.014 and p=0.005, respectively). (PDF 279 KB)

Supplementary file 5 Figure S5. The majority of CD45^+﻿^ immune cells present in digested tissue ended up in the filtrate. Different cellular fractions generated from organoid preparations of digested breast tissue (filtrate and organoid fractions) and human mammary epithelial cell (HMEC) cultures at passage 4 and 5 (p4, p5) were examined for CD45^+﻿^ expression (n=3 specimens, for each sample type). (PDF 13 KB)

Supplementary file 6 Figure S6. Density of both CD163^﻿+^ macrophage populations does not change with age. Density of CD163^+﻿^/CD68^-^ and CD163^+^/CD68^+^ macrophages quantified in situ from young (≤41y, n=8) and older (≥58y, n=9) donor age groups. Two-tailed, unpaired t-tests gave NS p-values for both populations. (PDF 37 KB)

Supplementary file 7 Figure S7. In silico immune signature analyses of normal breast tissue supported in situ results. Immune signature scores were calculated in publicly available gene expression data, GSE102088 (24), from normal bulk tissue reduction mammoplasties classified as either: young ≤35y (n=51) or older ≥50y (n=23). Signatures that significantly changed from the young to older age groups are shown here. Superscript indicates source of signature: ^1^Amara, et al., 2016, ^2^Bindea, et al., 2013, ^3^Danaher, et al., 2017, ^4^Senbabaoglu, et al., 2016. φ Both innate and adaptive immune cells are involved in the process of angiogenesis; this signature was placed in the adaptive section strictly for formatting purposes. (PDF 137 KB)

Supplementary file 8 Table S1. Details of published immune signatures used for in silico analyses of normal breast tissue. Results of immune signature scores calculated in GSE102088 (24) from normal bulk tissue reduction mammoplasties classified as either young ≤35y (n=51) or older ≥50y (n=23). Table shows all signatures that were included in analyses. “Change with age” column indicates whether transcriptional signature was upregulated or downregulated from young to older age group. Upregulated signatures are bolded to distinguish from downregulated signatures. Superscript indicates source of signature: ^1^Amara, et al., 2016, ^2^Bindea, et al., 2013, ^3^Danaher, et al., 2017, ^4^ Senbabaoglu, et al., 2016. (XLSX 22 KB)

## Data Availability

The publicly available gene expression data used in the present study can be accessed from the NCBI GEO database under accession number GSE102088 (24). All other raw data used and analyzed in this study are available from the corresponding author upon reasonable request.

## Abbreviations

BC: Breast cancer
IHC: Immunohistochemistry
BRCA1: Breast cancer gene 1
BRCA2: Breast cancer gene 2
HR: High risk
AR: Average risk
COH: City of Hope
FFPE: Formalin-fixed, paraffin-embedded
EER: Epithelium-enriched regions
FSR: Fat or stroma regions
PE: Peri-epithelium
ILS: Intralobular stroma
PBMCs: Peripheral blood mononuclear cells
RM: Reduction mammoplasty
P: Peripheral-to-tumor
C: Contralateral-to-tumor
PM: Prophylactic mastectomy
ANOVA: Analysis of variance
DAB: 3,3′-Diaminobenzidine
BMI: Body mass index
ER^+^: Estrogen receptor-positive
HER2^+^: Human epidermal growth factor receptor 2-positive
TNBC: Triple-negative breast cancer
