# Supplementary figures and images for: Changes in Immune Cell Types with Age in Breast are Consistent with a Decline in Immune Surveillance and Increased Immunosuppression
Source: J Mammary Gland Biol Neoplasia. 2021 Aug 2;26(3):247–61. doi: 10.1007/s10911-021-09495-2 (PMC8566425; doi:10.1007/s10911-021-09495-2)

A

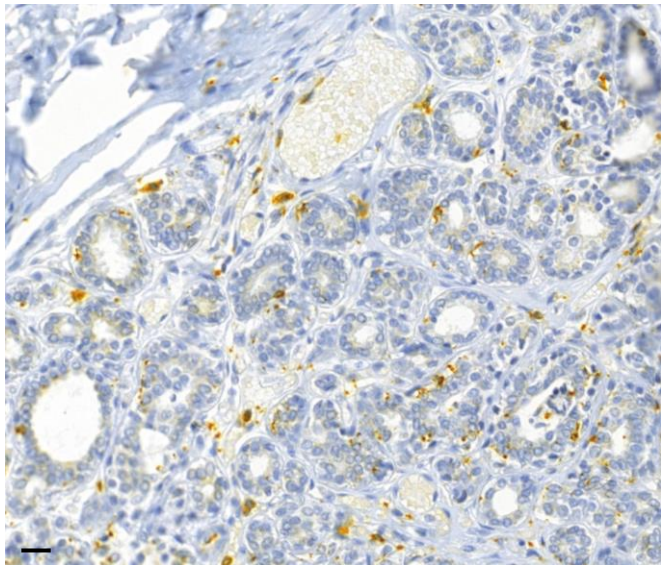

B

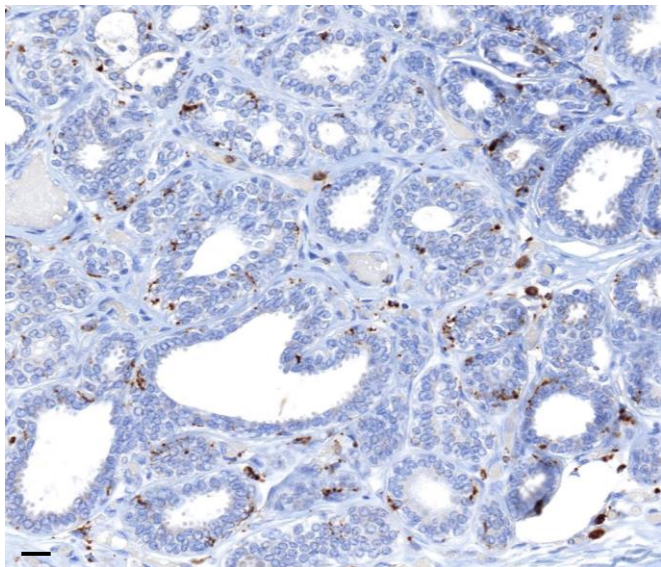

Supplement: Supplementary file 1 — Figure S1. 3,3’-Diaminobenzidine (DAB) staining confirmed yellow chromogen staining for CD68 via IHC. Adjacent tissue sections incubated with anti-CD68 monoclonal antibody were visualized with either (A) yellow chromogen or (B) traditional brown DAB chromogen. Scalebars are 20µm. (PDF 176 KB) [file 10911_2021_9495_MOESM1_ESM.pdf]

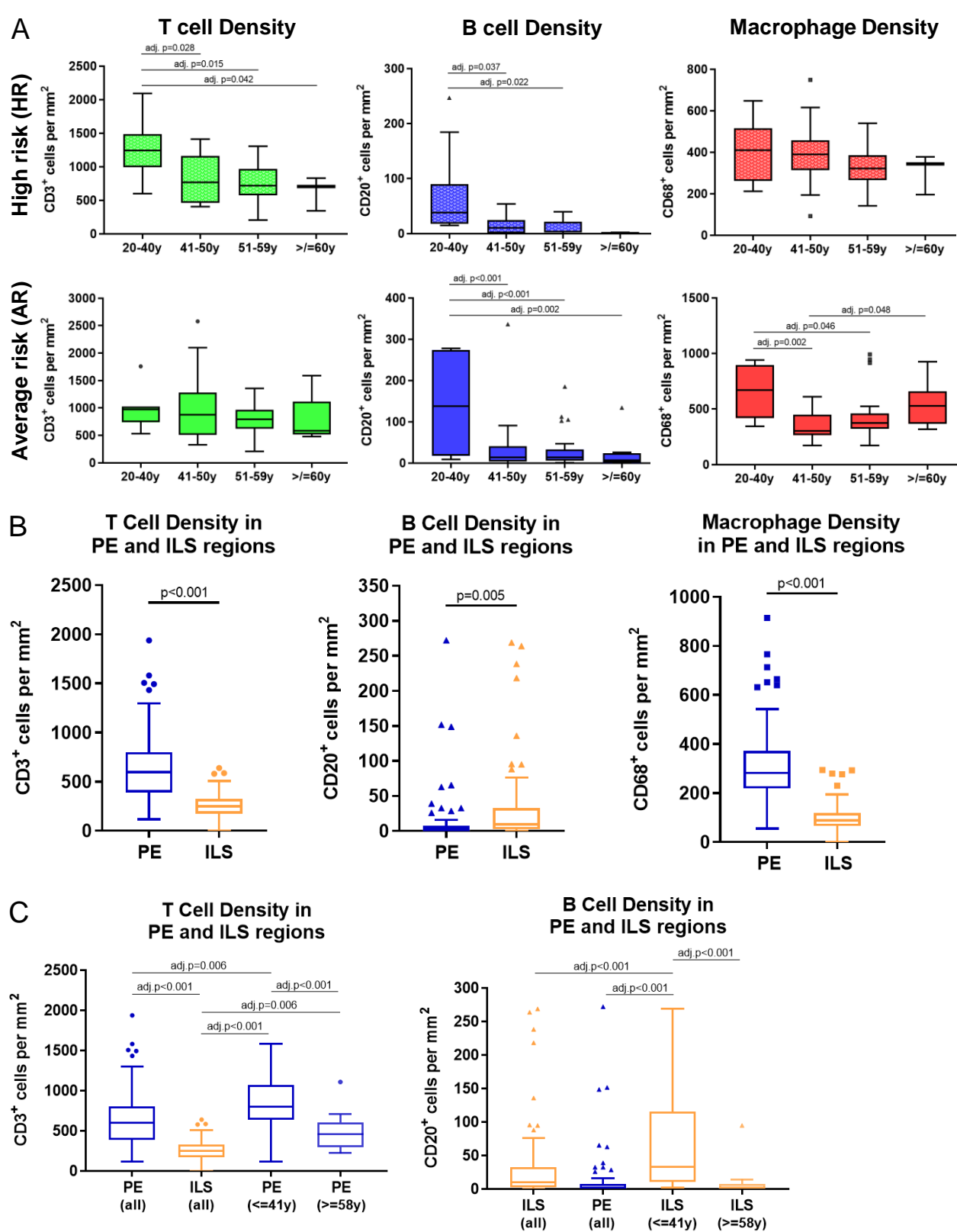

Supplement: Supplementary file 3 — Figure S3. Genetic risk status of tissue and proximity to mammary epithelium yields differences in immune cell densities. (A) ANOVA performed on IHC cell density data separated by BC genetic risk and age group showed age range may explain variations in T cell and B cell densities in HR tissues (p=0.006 and p=0.013, respectively) and variations in B cell and macrophage densities in AR tissues (p<0.001 and p=0.001, respectively). Adj. p-val of post-hoc analyses between age groups are indicated. 20-40y (HR: n=10, AR: n=7), 41-50y (HR: n=12, AR: n=27), 51-59y (HR: n=11, AR: n=24), ≥60y (HR: n=3, AR: n=9). (B) Immune cell densities (cells per mm2﻿) quantified in PE (blue) were compared to densities quantified in ILS and distant from the epithelium (orange) (n=102). T cells and macrophages had higher densities in PE and B cells had higher density in ILS. Two-tailed, paired t-test p-values are indicated. (C) Comparisons between T cell or B cell densities in PE and ILS in all (24-74y, n=102), young (≤41y, n=21), and older (≥58y, n=19) age groups show a decline in ILS B cell and PE T cell densities with age. Adj. p-val of post-hoc analyses are indicated. (PDF 200 KB) [file 10911_2021_9495_MOESM3_ESM.pdf]

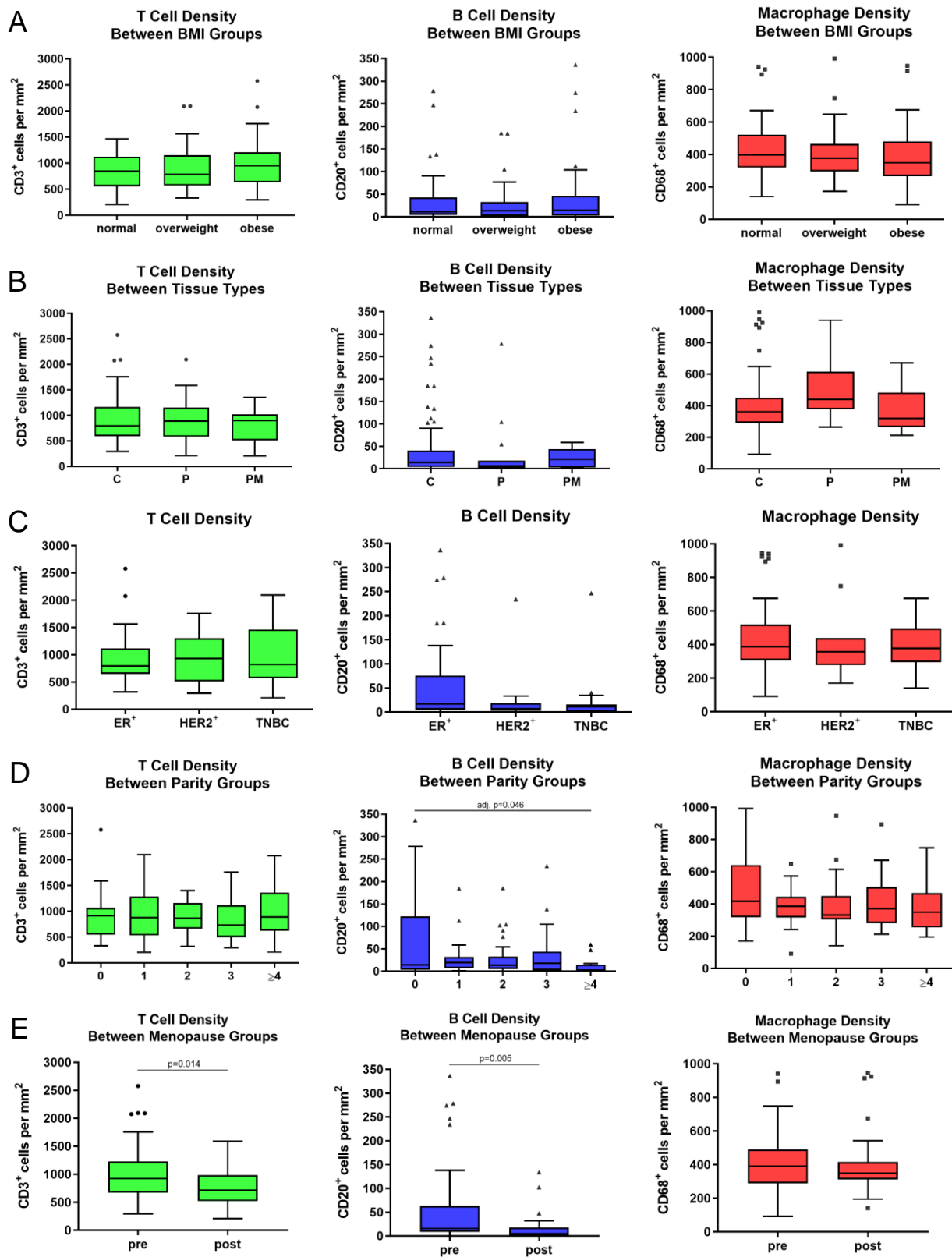

Supplement: Supplementary file 4 — Figure S4. Tissue samples grouped by other factors that may influence immune milieu and breast cancer risk. (A) ANOVA for immune cell type and BMI (n=102): normal weight (18.5 ≥ BMI < 25) (n=34), overweight (25 ≥ BMI < 30) (n=39), obese (BMI ≥ 30) (n=29). (B) ANOVA for immune cell type and tissue type (n=102): contralateral, C (n=77), peripheral, P (n=15), prophylactic mastectomy, PM (n=11). (C) ANOVA for immune cell type and receptor subtype of peripheral or contralateral tumor tissue (n=82): ER+﻿ (n=56), HER2+﻿ (n=11) triple-negative breast cancer, TNBC (n=15). (D) ANOVA for immune cell type and parity, defined as the number of pregnancies resulting in live births, (n=102): 0 (n=21), 1 (n=17), 2 (n=31), 3 (n=20), 4 or more (n=13). The only significant difference was seen in B cells (p=0.048) and post-hoc analysis indicated a difference between the parity groups 0 and ≥4 (adj. p=0.046). (E) Two-tailed, unpaired t-tests were used to compare immune cell densities between samples based on donor menopause status (n=93): pre (n=54), post (n=39). T cell and B cell densities decreased in post-menopause tissues (p=0.014 and p=0.005, respectively). (PDF 279 KB) [file 10911_2021_9495_MOESM4_ESM.pdf]

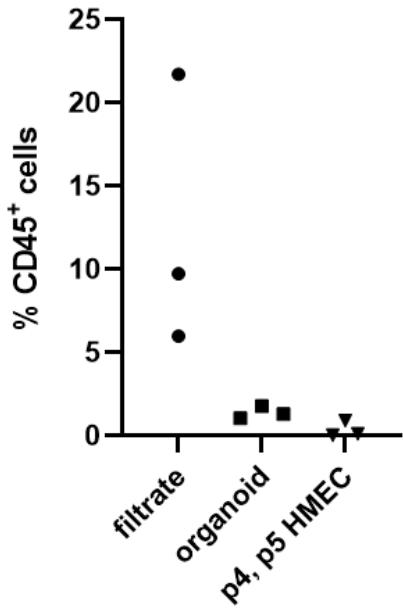

Supplement: Supplementary file 5 — Figure S5. The majority of CD45+﻿ immune cells present in digested tissue ended up in the filtrate. Different cellular fractions generated from organoid preparations of digested breast tissue (filtrate and organoid fractions) and human mammary epithelial cell (HMEC) cultures at passage 4 and 5 (p4, p5) were examined for CD45+﻿ expression (n=3 specimens, for each sample type). (PDF 13 KB) [file 10911_2021_9495_MOESM5_ESM.pdf]

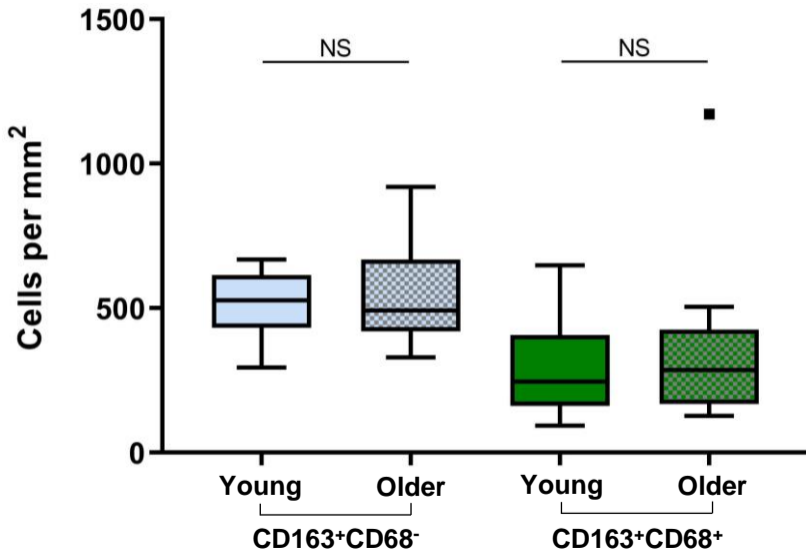

Supplement: Supplementary file 6 — Figure S6. Density of both CD163﻿+ macrophage populations does not change with age. Density of CD163+﻿/CD68- and CD163+/CD68+ macrophages quantified in situ from young (≤41y, n=8) and older (≥58y, n=9) donor age groups. Two-tailed, unpaired t-tests gave NS p-values for both populations. (PDF 37 KB) [file 10911_2021_9495_MOESM6_ESM.pdf]

## Innate Gene Signatures

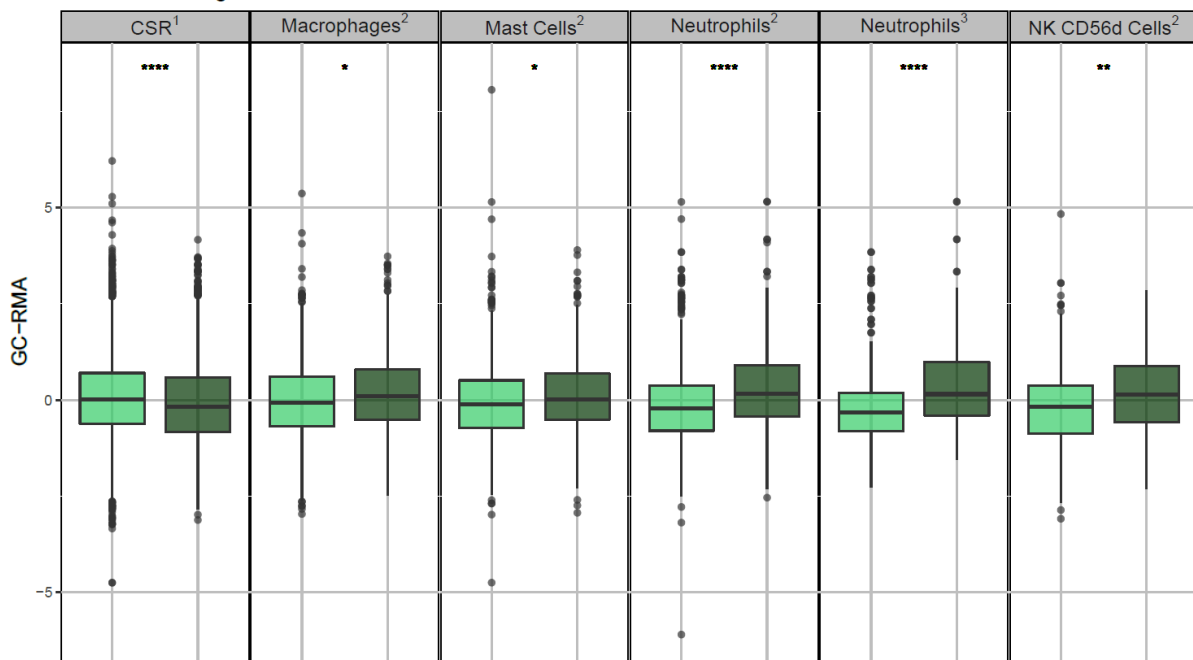

## Adaptive Gene Signatures

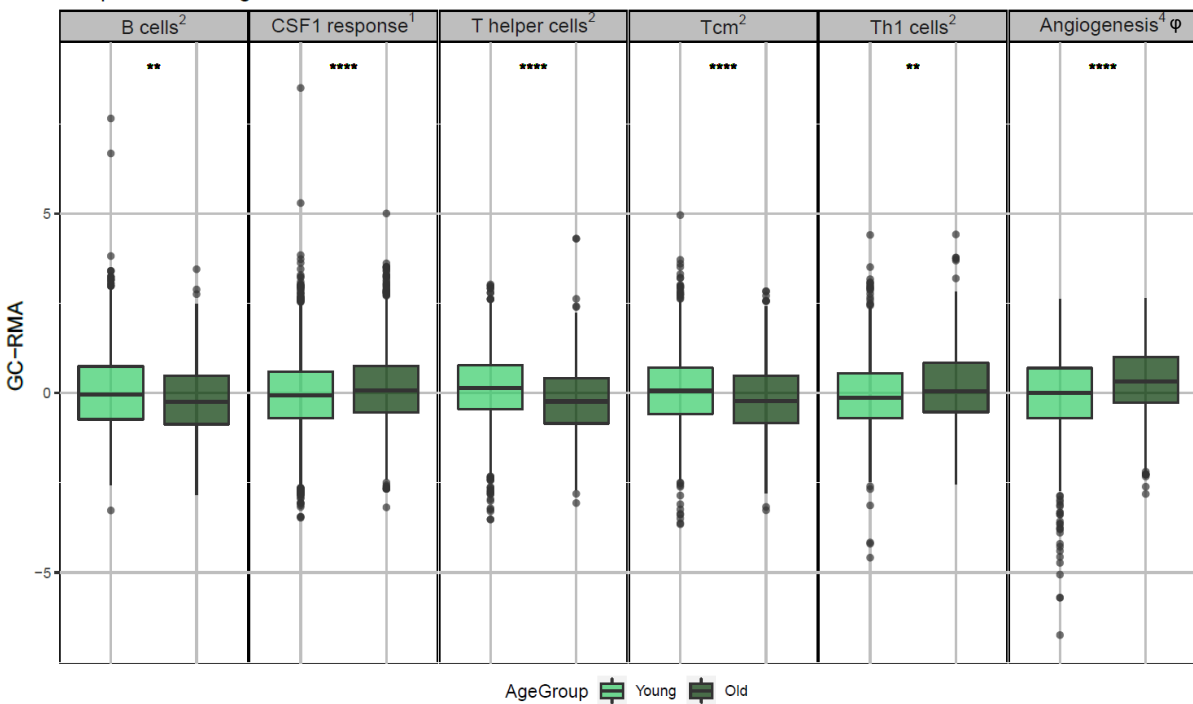

Supplement: Supplementary file 7 — Figure S7. In silico immune signature analyses of normal breast tissue supported in situ results. Immune signature scores were calculated in publicly available gene expression data, GSE102088 (24), from normal bulk tissue reduction mammoplasties classified as either: young ≤35y (n=51) or older ≥50y (n=23). Signatures that significantly changed from the young to older age groups are shown here. Superscript indicates source of signature: 1Amara, et al., 2016, 2Bindea, et al., 2013, 3Danaher, et al., 2017, 4Senbabaoglu, et al., 2016. φ Both innate and adaptive immune cells are involved in the process of angiogenesis; this signature was placed in the adaptive section strictly for formatting purposes. (PDF 137 KB) [file 10911_2021_9495_MOESM7_ESM.pdf]
