# Supplementary material for: Changes in Immune Cell Types with Age in Breast are Consistent with a Decline in Immune Surveillance and Increased Immunosuppression
Source: J Mammary Gland Biol Neoplasia. 2021 Aug 2;26(3):247–61. doi: 10.1007/s10911-021-09495-2 (PMC8566425; doi:10.1007/s10911-021-09495-2)
Supplement: Supplementary file 2 — Figure S2. Pipelines for training the u-net model in VisioPharm. (A) Pipeline for training the u-net model to classify epithelium-enriched regions (EER) and fat or stroma regions (FSR). (B) Pipeline for training the u-net model to classify peri-epithelium (PE) and intralobular stroma (ILS) within the EER. (PDF 126 KB) [file 10911_2021_9495_MOESM2_ESM.pdf]

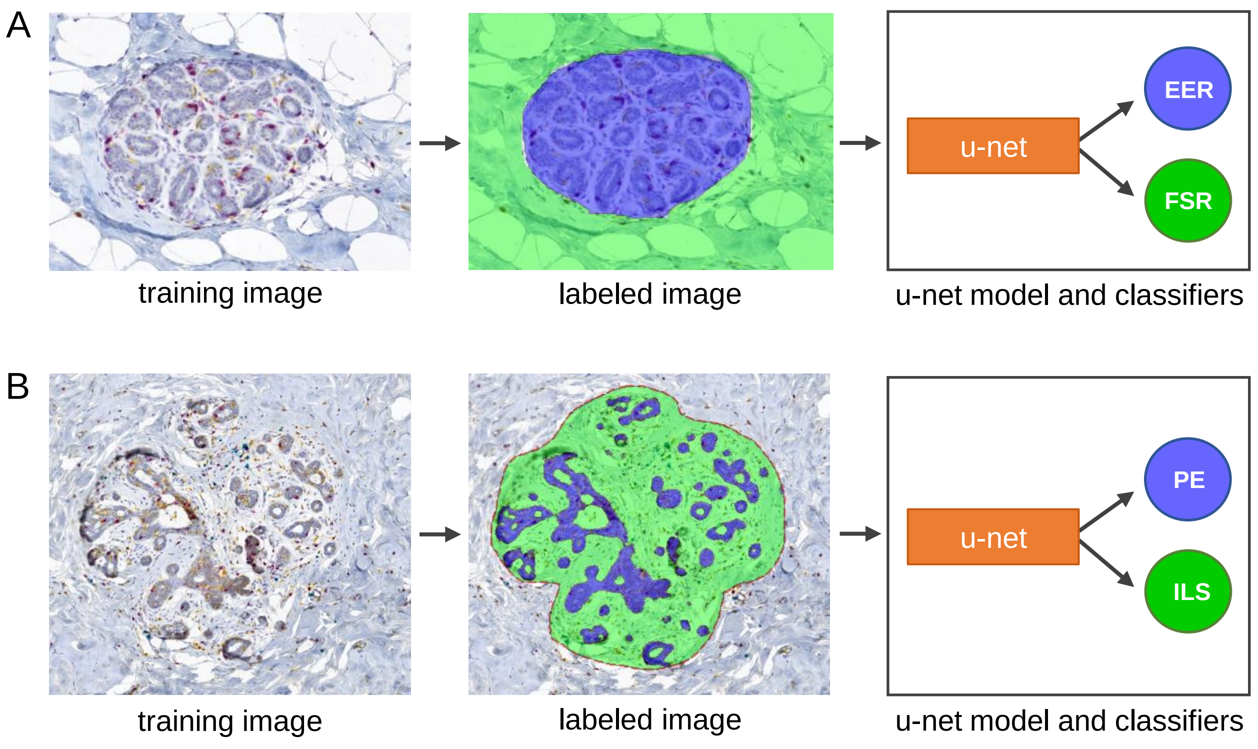
A
training image
labeled image
u-net
EER
FSR
u-net model and classifiers
B
training image
labeled image
u-net
PE
ILS
u-net model and classifiers
